# Production of stable bispecific IgG1 by controlled Fab-arm exchange: Scalability from bench to large-scale manufacturing by application of standard approaches

**DOI:** 10.4161/mabs.26233

**Published:** 2013-08-22

**Authors:** Michael J Gramer, Ewald TJ van den Bremer, Muriel D van Kampen, Amitava Kundu, Peter Kopfmann, Eric Etter, David Stinehelfer, Justin Long, Tom Lannom, Esther H Noordergraaf, Jolanda Gerritsen, Aran F Labrijn, Janine Schuurman, Patrick HC van Berkel, Paul WHI Parren

**Affiliations:** 1 Genmab; Brooklyn Park, MN USA; 2 Genmab; Utrecht, the Netherlands

**Keywords:** bispecific antibodies, scale up, large-scale manufacturing, cell culture, purification, controlled Fab-arm exchange

## Abstract

The manufacturing of bispecific antibodies can be challenging for a variety of reasons. For example, protein expression problems, stability issues, or the use of non-standard approaches for manufacturing can result in poor yield or poor facility fit. In this paper, we demonstrate the use of standard antibody platforms for large-scale manufacturing of bispecific IgG1 by controlled Fab-arm exchange. Two parental antibodies that each contain a single matched point mutation in the CH3 region were separately expressed in Chinese hamster ovary cells and manufactured at 1000 L scale using a platform fed-batch and purification process that was designed for standard antibody production. The bispecific antibody was generated by mixing the two parental molecules under controlled reducing conditions, resulting in efficient Fab-arm exchange of >95% at kg scale. The reductant was removed via diafiltration, resulting in spontaneous reoxidation of interchain disulfide bonds. Aside from the bispecific nature of the molecule, extensive characterization demonstrated that the IgG1 structural integrity was maintained, including function and stability. These results demonstrate the suitability of this bispecific IgG1 format for commercial-scale manufacturing using standard antibody manufacturing techniques.

## Introduction

The success of monoclonal antibodies (mAbs) as protein therapeutics has led the biopharmaceutical industry to invest heavily in high-yielding, robust manufacturing processes that facilitate rapid development and cost-effective production. As a result, platform approaches for both upstream cell culture and downstream purification have become well established in industry.^1^^,^^2^ These platform approaches accelerate the development of new mAbs by exploiting product similarities to standardize production and purification processes. Ideally, novel therapeutic formats (e.g., glyco-engineering, Fc-engineering, conjugation) should not affect critical product similarities so that manufacturing remains amenable to current platform approaches.

Bispecific antibodies (bsAbs), which contain two distinct binding specificities, are regarded as promising therapeutic agents, as evidenced by the abundance of bispecific formats in development.^3^ Early bsAb formats based on co-expression of relevant heavy (H) and light (L) chains^4^ or chemical crosslinking^5^ suffered from lack of product homogeneity, and the subsequent purification complexity resulted in poor product yield. Since then, multiple protein engineering strategies have enabled the design of formats with increased homogeneity and yield, either by improving the desired H-H and H-L pairing upon co-expression^6^^-^^11^ or by combining both antigen binding sites in a single polypeptide chain (or single HL pair).^12^^-^^18^ Although these strategies resolved some of the manufacturing issues, it was often at the expense of the physicochemical or pharmacokinetic (PK) properties of these agents.^19^^-^^22^

We recently described a method to generate stable bispecific IgG1 (bsIgG1) termed controlled Fab-arm exchange (cFAE).^23^ The method involves the separate expression of IgG1 mAbs that each contain a single matched point mutation at the CH3-CH3 domain interface. During controlled reduction of hinge disulfide bridges in vitro, the matched mutations drive the efficient recombination of binding arms. The process is essentially unidirectional because the mutations are selected to weaken the non-covalent CH3-CH3 interaction in the parental IgG1 mAbs, which results in dissociation of HL homodimers, and, at the same time, allows the formation of a strongly favored heterodimeric HL interaction. These characteristics strongly promote bsIgG1 end product yield and post-exchange stability upon reoxidation of the hinge. The use of a wild-type IgG1 hinge that is resistant to reduction under physiological conditions in vivo^24^ further adds to the post-exchange stability of the bsIgG1 end product.^23^

As reported here, we evaluated the production and purification of a model pair of parental mAbs, IgG1-K409R-CD20 and IgG1-F405L-EGFR, in our standard production platform at clinical manufacturing scale. We demonstrate robust scale-up of the cFAE process by applying the process developed at bench-scale using discontinuous diafiltration to clinical-scale production using a continuous, scalable diafiltration process. Structural and functional characterization, including stability, of the bsIgG1 product was assessed.

## Results

### Parental antibody manufacturing

To demonstrate that cFAE^23^ was compatible with large-scale manufacturing, the required quantities of IgG1-K409R-CD20 based on human mAb (HuMab) 7D8^25^ directed against the CD20 antigen, and IgG1-F405L-EGFR, based on HuMab 2F8^26^ directed against epidermal growth factor receptor (EGFR), were generated using recombinant Chinese hamster ovary (CHO) cell lines. Because the primary goal was the generation of sufficient material, abbreviated cell line development protocols were used (fewer cells screened, only one subcloning, cell line stability was not evaluated) and no process development was performed to optimize production. Instead, the best clone for each homodimer (based on productivity in a μ-24 micro reactor) was scaled up and inoculated into a 1000 L production bioreactor, and each homodimer was separately recovered and purified according to procedures that have worked well for standard antibodies.

Standard vectors, cell transfection techniques, and cell selection protocols were used to generate clones expressing each homodimer. Upon scale-up, cells grew as expected, with high viabilities (>90%) and doubling times of ~20 to 30 h for each cell line (data not shown). Cells grew well in each production bioreactor reaching a maximum cell density of 23–28 million viable cells/ml (Table 1). Each homodimer was recovered from the bioreactor by centrifugation followed by filtration. The IgG1-K409R-CD20 bioreactor was harvested on day 11 since sufficient material had been produced, whereas the IgG1-F405L-EGFR bioreactor run was extended to 15 d due to relatively lower productivity (Table 1). The entire process of cell transfection through bioreactor harvest was in line with expectation for production of standard antibodies, indicating that the point mutation for each homodimer was compatible with commercial upstream manufacturing approaches.

**Table:** **Table 1.** Summary of homodimer bioreactor production and harvest

Homodimer	Maximum Viable Cell Density (million/ml)	Run Time (days)	Overall Specific Productivity (pg/cell/day)	Final Viability (%)	Final Titer (g/L)	Final Volume (L)	Total Produced (g)	Harvest Recovery (%)
IgG1-K409R-CD20	28	11	15.5	87	1.85	903	1671	93
IgG1-F405L-EGFR	23	15	4.4	52	0.60	1114	668	80

To assess compatibility with routine downstream platform processes developed for large-scale production of IgG1, the parental mAbs were purified using standard protein A chromatography, low pH viral inactivation, and anion-exchange chromatography. An ultrafiltration/diafiltration (UF/DF) step was employed as the final unit operation to formulate each of the parental mAbs into a phosphate buffered saline (PBS) pH 7.4 buffer previously demonstrated to be compatible with cFAE.^23^ In each case, purification process performance was in line with expectation based on standard antibodies, including high yields, low aggregate levels, and low residual process-related impurities (Tables 2 and 3).

**Table:** **Table 2.** Homodimer purification yield

Homodimer	Yield Across Protein A and VI (%)	Yield Across AEX (%)	Yield Across UF/DF (%)	Overall Yield Unprocessed Bulk to Final Material (%)	Final Concentration (g/L)
IgG1-K409R-CD20	90.4	96.4	98.5	79.8	26.2
IgG1-F405L-EGFR	95.5	96.0	100.3	73.6	28.0

**Table:** **Table 3.** Homodimer impurity clearance

Process Step	IgG1-K409R-CD20				IgG1-F405L-EGFR			
	Monomer by SEC (%)	HCP (ng/mg)	DNA (pg/mL)	Protein A (ng/mg)	Monomer by SEC (%)	HCP (ng/mg)	DNA (pg/mL)	Protein A (ng/mg)
Cell-free harvest	-	372 090	not determined	-	-	3 339 875	not determined	-
Post VI pool	99.9	16	not determined	0.72	99.2	1098	not determined	1.82
Post UF/DF final product	100.0	0.5	< 10	< 0.16	99.7	1.3	< 10	< 0.16

### Generation of bsIgG1-EGFRxCD20 at manufacturing-scale by cFAE

The cFAE process was evaluated at 25 L scale using 10 g/L of each parental antibody in PBS pH 7.4 to create a 0.5 kg batch; a 5 ml (100 mg) bench-scale exchange was similarly performed as a reference (Table 4). The cFAE process was initiated by addition of 50 mM 2-mercaptoethylamine•HCl (2-MEA), which was removed after 5 h by diafiltration against PBS pH 7.4. Diafiltration was performed at bench-scale using a semi-batch centrifugal ultrafiltration unit and at manufacturing-scale using a standard continuous flow diafiltration process (Fig. 1). Samples were taken for in-process characterization of the exchange reaction during both the reduction phase and the diafiltration (re-oxidation) phase. A sample aliquot was taken and the reaction was immediately quenched using iodoacetamide (IAA) and analyzed by SDS-PAGE to determine the extent of interchain disulfide bond linkage. The remainder of each sample was analyzed by analytical CIEX chromatography to assess the extent of cFAE. CIEX is highly suited for analysis of this cFAE process because the difference in charge between the parental antibodies and the bispecific product, with an intermediate charge profile, was sufficiently different to obtain good separation (Fig. 2).

**Table:** **Table 4.** Process summary

Process parameter	20 g/L bench- scale process	20 g/L manufacturing-scalep
Total volume	5 mL	25 L
IgG1-F405L-EGFR	10 g/L	10 g/L
IgG1-K409R-CD20	10 g/L	10 g/L
Temperature	18–22 °C	22–24 °C
2-MEA	50 mM	50 mM
Reduction time	5 h	5 h
2-MEA removal	Vivaspin 6 filter	Diafiltration
Lot size	100 mg	500 g

**Figure:**
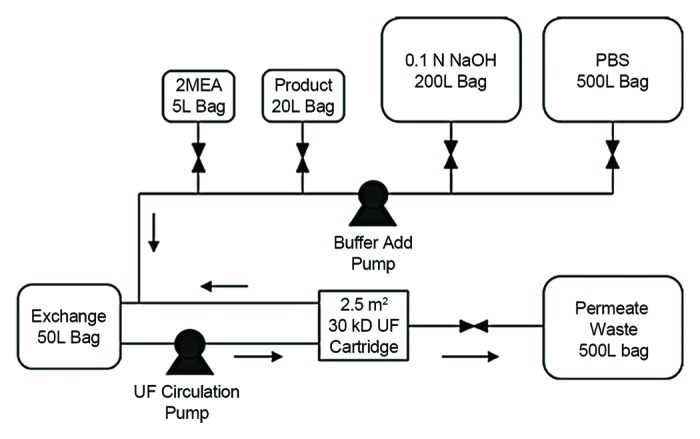
**Figure 1.** Process flow diagram for the disposable exchange reaction system at manufacturing-scale. The 50 L bag-UF cartridge loop was first sanitized with 0.1 N NaOH from the 200 L NaOH bag. The system was then flushed with PBS from the 500L bag to bring the pH to 7.4. The homodimers (250 g each in 18.48 L PBS) were added from the 20 L product bag and additional PBS was added from the 500 L bag (to 20.83 L total volume). The reaction was initiated by adding 300 mM 2-MEA (4.17 L) from the 5 L 2-MEA bag, and the system was circulated for 5 h. The 2-MEA was then washed out via constant volume diafilration using PBS from the 500 L bag.

**Figure:**
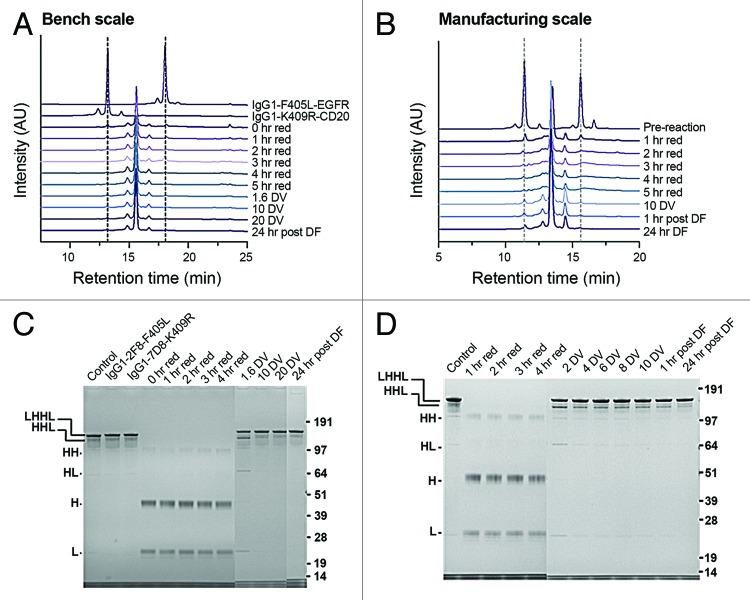
**Figure 2.** In process characterization by CIEX (**A** and **C**) and SDS-PAGE (**B** and **D**) at bench-scale (**A, B**) and manufacturing-scale (**C **and** D**). Retention times in the CIEX elution profiles in panels (**A**) and (**C**) are slightly different due to different analytical systems used at the research and manufacturing site. Furthermore, injection amounts differ (i.e., 50 µg at bench-scale (**A** and **B**) vs. 100 µg manufacturing-scale (**C** and **D**).

SDS-PAGE analysis demonstrated almost complete reduction of both HH and HL interchain disulfide bonds within the first sampling time point of 1 h (Fig. 2). Upon diafiltration, the molecule started re-oxidizing, with the extent of re-oxidation increasing with increasing removal of the reductant. The molecule was completely re-oxidized by the end of diafiltration.

CIEX analysis demonstrated efficient exchange of the parental mAbs into the heterodimer product (Fig. 2); substantial exchange had already occurred within the first sampling time point at 1 h reduction and was complete by 2–3 h as indicated by the decrease of the parental antibody peaks. A number of intermediate forms were apparent in low abundance; these became less apparent during the course of diafiltration. The final extent of cFAE was > 95% as determined by CIEX for both the bench and manufacturing-scale processes (Table 5). Determination of the concentration of the beginning and final samples by OD280 demonstrated complete recovery for the manufacturing-scale process (Table 5). Furthermore, HP-SEC of the final sample demonstrated that the process did not induce product aggregation (> 99% monomer) and residual 2-MEA concentrations were low in both cases (Table 5). These results demonstrate that the cFAE process can be efficiently scaled up to meet manufacturing requirements.

**Table:** **Table 5.** Extent of exchange, recovery and aggregation, and residual reductant following cFAE

Condition	% by CIEX			Total Protein by A280 (g/L)	% by HP-SEC			Residual 2-MEA (µM)
	IgG1-F405L-EGFR	bsIgG1-EGFRxCD20	IgG1-K409R-CD20		Mon	Agg	Deg	
20 g/L; 5 mL	3.5	95.0	1.6	ND	99.4	0.6	< 0.1	20.5 ± 1.2
20 g/L; 25 L	2.8	96.3	0.9	20.0	99.4	0.6	< 0.1	2.9 ± 0.6

ND, not determined; Mon, monomer; Agg, aggregate; Deg, degradation

### Structural and functional characterization

First, functional (dual) binding properties were assessed for bsIgG1-EGFRxCD20 (Fig. 3). The results showed that the large-scale produced bsIgG1-EGFRxCD20 showed similar dual-binding properties compared with a bench-scale produced bsIgG1-EGFRxCD20 reference. The parental homodimers showed no dual-binding properties as expected.

**Figure:**
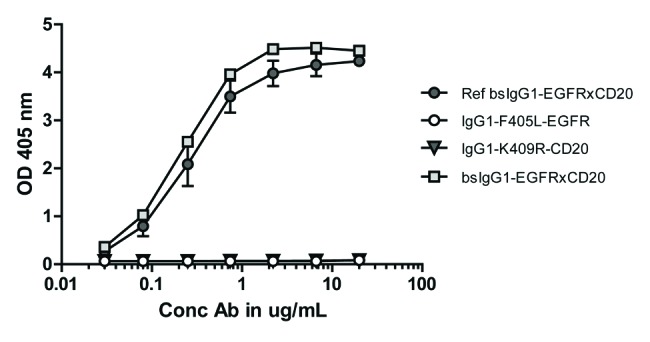
**Figure 3.** Dual-binding activity of bsIgG1-EGFRxCD20 prepared at large-scale, compared with a reference prepared at bench scale and the two parental homodimers bsIgG1-EGFRxCD20. Data represent mean ± SEM.

Next, ESI-MS analyses on intact deglycosylated IgG1-F405L-EGFR, IgG1-K409R-CD20 and bsIgG1-EGFRxCD20 were performed to verify the exact mass integrity (Fig. 4). The theoretical masses for IgG1-F405L-EGFR (146,287 Da) and IgG1-K409R-CD20 (146 026 Da) corresponded well with the measured masses that were detected at 146 290 Da (i.e., ∆ mass +3Da; measured minus theoretical) and 146,027 Da (i.e., ∆ mass +1 Da), respectively, indicating that both parental mAbs were correctly prepared. For bsIgG1-EGFRxCD20, a mass of 146 159 Da was obtained for both production scales, which was in agreement with the theoretical mass for the heterodimeric bsAb, i.e., 146 158 Da (∆ mass + 1 Da). Upon reduction of bsIgG1-EGFRxCD20, all four chains, i.e., heavy chains and light chains of both parental mAbs, appeared in each MS spectrum as expected (data not shown).

**Figure:**
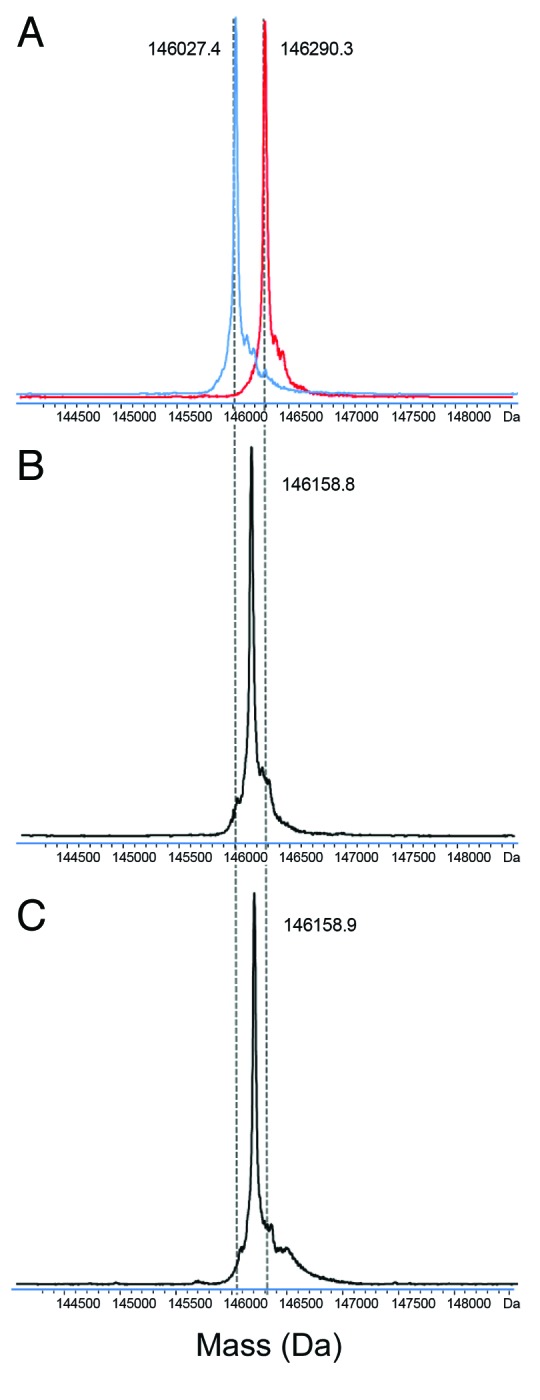
**Figure 4.** ESI-MS spectra of deglycosylated homodimers IgG1-F405L-EGFR (red trace) and IgG1-K409R-CD20 (blue trace) (**A**). Bispecific antibody bsIgG1-EGFRxCD20 produced at 25 L (**B**) and 5 mL (**C**) scale starting with mixture with each homodimer at 10 g/L.

Because a single point mutation in the CH3 domain of IgG1 and IgG4 can dramatically alter the N-linked glycan structure in the CH2 domain,^27^ and changes in the N-linked glycosylation may impact clinical efficacy,^28^ the N-linked glycan structures of both parental mAbs containing the F405L or K409R point mutations were analyzed. For this, N-linked glycan profiles for IgG1-F405L-EGFR, IgG1-K409R-CD20 and bsIgG1-EGFRxCD20 were generated by 2-aminobenzamide (2-AB)-labeled N-glycans in conjunction with normal-phase chromatography (Table 6). All obtained N-linked glycan profiles were similar to those observed for regular IgGs,^28^^,^^29^ with G0F glycan dominating the profiles. For the bsIgG1, glycan values were obtained that reflect the average of both parental mAbs. No uncommon N-linked glycans were detected.

**Table:** **Table 6.** Summary of 2-AB N-linked glycan analysis of the parental antibodies and the bsIgG1 produced at manufacturing-scale

Antibody	% G0	% G0F	% Man-5	% G1F	% G2F	% Galact.	% Fucosylation
IgG1-F405L-EGFR	2.4	88.3	3.7	0.3	3.1	2.2	96.7
IgG1-K409R-CD20	1.5	87.5	0.5	ND	9.1	5.2	98.2
bsIgG1-EGFRxCD20	2.1	88.4	2.2	0.2	5.5	3.0	97.6

ND, not detected

To investigate potential changes in secondary structure that occurred as a result of the point mutations, circular dichroism (CD) measurements were performed (Fig. 5). The results obtained for the far-UV CD spectra showed the characteristic shape expected for IgG molecules, with a strong negative ellipticity mainly in the region of 215 to 220 nm deriving from β-sheet structures. While there was no substantial difference in the secondary structure observed, some (small) differences in the near-UV CD spectra between the two parental mAbs were observed. Differences in the near-UV region reflect differences in the tertiary structure and folding of the protein. The shape of the near-UV CD spectra, however, was also as typically measured for IgG.^30^ The differences observed were likely related to the Fab part because the corresponding wild-type IgG1s showed similar spectra. Together, these data show that no substantial structural changes were induced upon mutating the F405 and K409 positions to leucine and arginine, respectively, in the CH3 domains. Interestingly, bsIgG1-EGFRxCD20 showed average ellipticity signals in the near-UV CD spectra, indicating that the structural properties of both parental mAbs were adopted by the bispecific end product.

**Figure:**
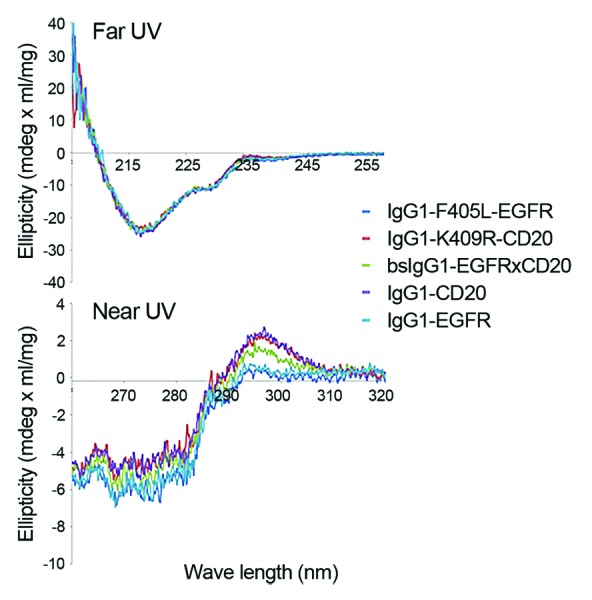
**Figure 5.** Far- and near-UV CD spectra of IgG1-F405L-EGFR, IgG-K409R-CD20 and bsIgG1-EGFRxCD20, wild-type IgG1-CD20 and wild-type IgG1-EGFR.

### Real time stability assessment

The stability of the large-scale bsIgG1-EGFRxCD20 end product upon storage in PBS (pH 7.4) has been described previously.^23^ In that work, we demonstrated no detectable changes in product quality over a period of 6 mo at 5 °C, with some expected degradation of the material observed when it was stored at 25 °C. The follow-up studies described here showed that the stability of bsIgG1-EGFRxCD20 could be further increased by formulation in slightly acidic pH buffers, resulting in reduced fragmentation and reduced deamidation at the higher temperature (i.e., from ~45% at pH 7.4 to ~20% acidic peaks at pH 6.0; Fig. S1‒S3).

In addition, we assessed the stability of bsIgG1-EGFRxCD20 at different pH in comparison to both parental antibodies and to a non-mutated wild-type IgG (IgG1-EGFR). The antibodies were exposed to high pH (Tris pH 8.5), low pH (acetate pH 4.0) and neutral pH (PBS pH 7.4) over 8 weeks. Although the magnitude of the effect depended on the storage condition, fragmentation as measured by HP-SEC under all conditions was clearly increased for IgG1-F405L-EGFR and wild-type IgG1-EGFR compared with IgG1-K409R-CD20 and the bsIgG1-EGFRxCD20 antibody (Fig. 6). The IgG1-EGFR wild-type showed a small increase in multimer level in PBS pH 7.4 (i.e., 0.5%) and Tris pH 8.5 (i.e., 1.0%), whereas aggregation was not observed for the other antibodies, including the bsIgG1, under these conditions. CIEX analysis revealed an acidic shift in the isoform distribution for all the antibodies upon incubation in PBS (pH 7.4) at 25 °C, and this shift was accelerated at pH 8.5 (Fig. 7). The IgG1-EGFR antibody, which was produced two years prior to this study, already contained a relatively high percentage of acidic peaks at the start of the study. Nevertheless, it can be concluded that the order in which the four antibodies were affected at both pH conditions was comparable, further implying that the mechanism was likely deamidation for both conditions. Storage in acetate buffer (pH 4.0) resulted in a decrease in neutral isoforms for all antibodies that was accompanied mainly by an increase in the basic isoforms. Such basic shifts could occur as a result of dehydration of the aspartate residues to a succinimide intermediate, which is favored under mildly acidic conditions as described previously for an IgG1.^31^ In summary, in all buffer solutions, the primary mode of degradation was through fragmentation. The results showed that the F405L and K409R mutations did not significantly affect degradation patterns of the homodimers compared with a wild-type IgG1 antibody. The bsIgG1, furthermore, showed a favorable pattern where it followed that of the IgG1-K409R-CD20 parent with less degradation compared with both the IgG1-F405L-EGFR parent, as well as the wild-type IgG1-EGFR antibody. The subtle differences observed between the stability of the parental antibodies likely were Fab sequence-dependent and did not result from the F405L and K409R mutations. Hence, the bispecific antibody showed no deviating behavior compared with the parental antibodies under the stressed conditions applied.

**Figure:**
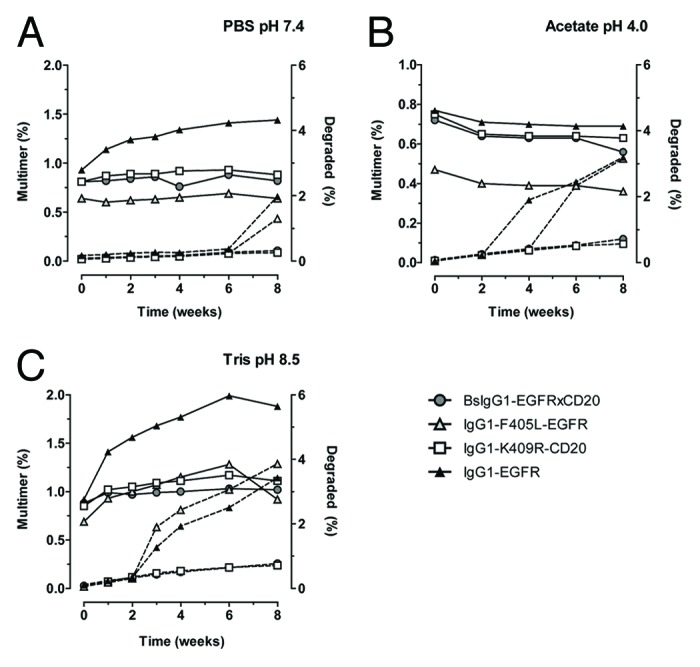
**Figure 6.** Stability of bsIgG1-EGFRxCD20, homodimers, and wild-type IgG1-EGFR as analyzed by HP-SEC. Multimers (left axis, solid line) and degradation (right axis; dotted line) in percentage (%) for the indicated mAbs upon 8 weeks storage at 25°C in (**A**) PBS pH 7.4; (**B**) Acetate pH 4.0; (**C**) Tris pH 8.5

**Figure:**
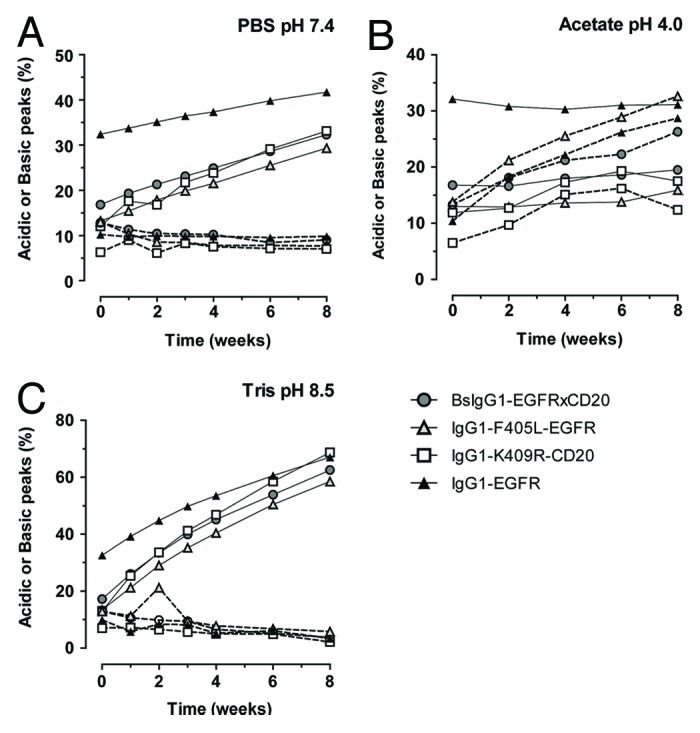
**Figure 7.** Stability of bsIgG1-EGFRxCD20, homodimers and wild-type IgG1-EGFR as analyzed by CIEX. Acidic peaks (solid line) and Basic peaks (dotted line) in percentage (%) for the indicated mAbs upon 8 weeks storage at 25°C in (**A**) PBS pH 7.4; (**B**) Acetate pH 4.0, and (**C**) Tris pH 8.5.

## Discussion

Due to elevated interest in the therapeutic potential of bispecific antibodies, a plethora of formats have been developed to exploit the promise of dual targeting antibodies.^3^ Very few formats, however, offer the flexibility to leverage standard antibody discovery and production methods while retaining regular IgG1 structure, function and stability. Therefore, the cFAE format described in this paper, and commercialized as the Duobody™ platform, is enabling in these aspects. Standard antibodies against any single target generated using established antibody discovery techniques can be applied to the cFAE bispecific format by their expression as a human IgG1 antibody containing an appropriate single point mutation in the CH3 domain to create a parental homodimer. Each parental mAb candidate is then expressed separately using standard antibody production and purification methods either in small-scale format for discovery or at large-scale for clinical application as demonstrated here. The cFAE process employs mildly reducing conditions followed by removal of the reductant and spontaneous re-oxidation using diafiltration, which is a scalable unit operation that is commonly incorporated into standard antibody production processes. These aspects enable this format to be seamlessly incorporated into mature antibody discovery, development, and manufacturing platforms.

Incubation of wild-type IgG1 antibodies in the presence of reductant does not result in exchange due to their relatively strong non-covalent CH3-CH3 interactions.^32^ Likewise, despite the fact that H-L interchain disulfide bonds are reduced during the cFAE process, L chain swapping was not detected at the resolution of the methods used.^23^ The incorporation of the matched point mutations alters the CH3-CH3 interactions to highly favor H chain heterodimerization while the L chains remain associated with the original H chain.

Analytical characterization and stability analysis combined with demonstration of expected antigen-binding characteristics, regular effector function and PK as previously shown^23^ demonstrates that the parental molecules and the bispecific antibody retain normal IgG1 structure, function and stability. Interestingly, in many biophysical characterization assays, the bispecific antibody characteristics lie between that of the two parental mAbs. In the case of the model pair used in this study, the physical characteristics were sufficiently unique to distinguish between the two parental molecules and the resulting bispecific antibody. For other antibody pairs, alternative analytical approaches may be required.

Transfection, selection, and growth profiles for cells expressing IgG1-F405L-EGFR and IgG1-K409R-CD20 were normal, demonstrating that the point mutations did not adversely affect these aspects. Although product expression and recovery were good, the cell-specific antibody secretion rate of parental homodimers was approximately 2- to 4-fold lower than we typically observe for our platform process (Table 1).^29^ The most likely explanation is that we used an abbreviated cell line development process without extensive selection for the highest producers to generate this proof-of-concept pair. The behavior of the homodimers in our downstream platform process was typical of what we observe for wild-type antibodies including high yield, good impurity clearance, and low aggregate formation (Tables 2 and 3). The cFAE process resulted in >95% conversion of homodimers into the bsIgG1. Depending on the target, antibody pair, mechanism of action, expected toxicity and indication, further downstream polishing may be required to remove residual homodimers. For the pair described in this paper, we achieved baseline separation by analytical CIEX (Fig. 2) which preliminary results suggest can be extended to preparative scale CIEX chromatography (data not shown). However, since the nature of any homodimer pair may vary quite significantly, CIEX is not likely to be generally applicable, in which case a number of commercially-viable other chemistries (e.g., HIC, CHT, mixed-mode resins) could be combined with chromatographic screening approaches to optimize the polishing approach. Furthermore, homodimer removal should be part of a developability/manufacturability assessment to evaluate potential purification issues during lead candidate selection.

The controlled Fab-arm exchange process, including incubation of the parental antibodies with a reductant followed by removal via diafiltration, was scalable from the milligram to kilogram scale both in extent of exchange and in resulting product quality (Tables 5 and 6; Fig. 2–7). Time, temperature, reductant type, and reductant concentration were previously demonstrated to affect the exchange process.^23^ Further process ranging for these and other potential factors that may impact the process are being investigated and defined.

Further scale-up for high quality, large-scale production of bispecific antibodies will be straightforward because standard upstream and downstream processes were used. In this study, a 0.5 kg lot of the bispecific product was generated in a reaction volume of 25 L. Since diafiltration is well-understood, robust, and scalable to thousands of liters, diafiltration can be leveraged for further scale-up of the cFAE process for batch sizes of tens to hundreds of kilograms.

## Materials and Methods

### Cell line development (abbreviated protocol)

Expression vectors for IgG1-K409R-CD20 and IgG1-F405L-EGFR have been described previously.^23^^,^^33^ Cell lines were generated by transfecting relevant heavy and light chain expression vectors into CHO-K1SV cells using the glutamine synthetase expression system (Lonza). Transfected cells were selected via ClonePix (Genetix) based on colony size and antibody staining. Twenty-four colonies for each parental homodimer were pooled, subcloned in 96-well plates, and screened for antibody titer. The top 12 clones for each antibody were screened in a μ-24 microreactor (Pall). The highest producer for each homodimer was chosen for large-scale production.

### Parental antibody production

For large-scale production, the previously described chemically-defined, amino acid-optimized fed-batch process (not including the uridine, manganese chloride, and galactose supplement) was used as the platform for 1000 L scale production,^29^ though some modifications were made based on in-process data. Specifically, the feed rate was adjusted according to glucose demand.

### Bioreactor harvest

At the end of each production run, the bioreactor was chilled to 12 °C and the unprocessed bulk was adjusted to pH 4.5 using 2 M citric acid to enhance particulate flocculation. After 45 min, the unprocessed bulk was harvested through a continuous flow disc-stack centrifuge followed by depth filtration and 0.2 micron filtration. The cell-free harvest was adjusted back to pH 6.5 using 2 M Tris base and held at 8 °C until purification.

### Parental antibody purification

All purification steps were performed at ambient temperature. The cell-free harvest was loaded on to a MabSelectSuRe protein A resin (GE Healthcare), washed with a pH 7.0 citrate buffer, and eluted in a pH 3.5 citrate buffer. The product was collected based on UV absorbance of the column effluent at 280 nm wavelength. The collected product was adjusted to pH 3.6 using 0.5 M HCl, held for 60 min for viral inactivation (VI), and adjusted to pH 7.8 using a 2 M Tris base buffer. Two protein A cycles were performed for each antibody. The pooled post VI material was loaded onto a Q Sepharose Fast Flow anion exchange column (GE Healthcare) and the column was washed using a pH 7.8 Tris buffer. This column was operated in a flow-through mode, thus process- and product-related impurities removed by this step were left bound to the column while the product was collected using UV absorbance at 280 nm wavelength. The collected material was concentrated to >25 g/L and buffer exchanged into PBS pH 7.4 (1.92 mM NaH_2_PO_4_•H_2_0, 8.66 mM Na_2_HPO_4_•7H_2_O, 140.31 mM NaCl) using a 2.5 m^2^ Omega 30 kDa ultrafiltration cartridge (Pall). The final homodimer material was dispensed into a disposable gamma irradiated Flexboy bag (Sartorius Stedim Biotech) through a 0.2 µm sterilizing grade filter and stored at 2–8°C.

### Bench-scale controlled fab-arm exchange

The cFAE reaction was initiated by adding a 300 mM stock solution of 2-MEA (Sigma/Fluka PN 30078) in PBS pH 7.4 to an equimolar mixture of IgG1-K409R-CD20 and IgG1-F405L-EGFR parental mAbs. The final solution contained 50 mM reductant and 10 mg/ml of each homodimer in 5 ml PBS pH 7.4. The samples were incubated for 5 h at room temperature (18–22 °C) and subsequently filtered by a Vivaspin 6 via centrifugal operation (Sartorius Stedim VS0602) to remove 2-MEA. At different time points during the incubation and subsequent filtration steps, two samples were taken (100 µl each). One sample was quenched with iodoacetamide (IAA) and stored at 5 °C for later analysis by SDS-PAGE, the other sample was immediately snap frozen and thawed to 5 °C prior to analytical CIEX analysis.

### Manufacturing-scale controlled Fab-arm exchange

The process flow diagram for the disposable exchange reaction system at manufacturing-scale is shown in Figure 1. All bags were from Sartorius Stedim. The 5 L, 20 L and 50 L bags were multilayer construction with an ethylene vinyl acetate inner layer and an ethylene vinyl alcohol outer layer. The 200 L and 500 L bags were multilayer construction with an ultra-low density polyethylene inner layer and an ethylene vinyl alcohol outer layer. All tubing was platinum cured silicone (Masterflex). Connections were made using Kleenpak connectors (Pall). A manual clamp was used to isolate each bag from the system. The buffer addition and circulation pumps were peristaltic pumps (Watson-Marlowe). A 2.5 m^2^ Pall Omega PES diafiltration cartridge was used (Pall). The reaction bag was placed on a wave mixer (GE Healthcare) set with a rock angle of 7° and a rocking rate of 30 per min. Scales were used to monitor bag weights.

Prior to the exchange reaction, the system was sanitized using a 0.1 M NaOH solution and then rinsed with PBS and drained. The morning of the exchange reaction, IgG1-K409R-CD20 and IgG1-F405L-EGFR were added in a 1:1 mass ratio by gravity feed (18.48 L total with 250 g of each parental antibody). PBS pH 7.4 was then added to bring the volume to 20.83 L and the system circulated at 7 LPM to mix the contents. The reaction was initiated by gravity addition of the 4.17 L 300 mM 2-MEA stock solution in PBS while maintaining circulation. After a 5 h incubation, the permeate valve was opened and the pump speed increased to meet a target inlet feed pressure of 10 PSI for diafiltration. The pump was set at 140 RPM (26 LPM), resulting in permeate rate of 2.1 L/min. The PBS addition path was opened and the buffer add pump speed was controlled to keep a constant weight in the reactor bag. Once the target diafiltration volume (DV) was collected in the 500 L waste bag (10 DV), the permeate valve was closed and the buffer add pump was stopped. The circulation pump was returned to 30 RPM (7 LPM) during the oxidation time. After overnight incubation, a second diafiltration of 4 DV was performed. All processes were performed at ambient temperature (22–24 °C). Samples were withdrawn from the system at various points throughout each run; a 400 µL portion of each sample was quenched with 8 µL IAA (1 M) (Sigma-Aldrich) for later analysis by SDS-PAGE, while the remaining sample was analyzed immediately by CIEX chromatography.

### Residual protein A assay

The quantification of residual leached MabSelect SuRe protein A ligand was performed by utilizing a Cygnus technologies Protein A kit. The 8-well microtiter strips are coated with polyclonal chicken anti-protein A. A standard curve concentration of 10 ng/mL to 0.078 ng/mL was used. Samples were initially diluted to 2 mg/mL protein concentration with a PBS-Tween-20 diluent followed by further in-plate dilution to 0.5 mg/mL with an acetate dissociation buffer. The samples were then incubated for 1 h at room temperature. Next, addition of anti-protein A conjugated to horseradish peroxidase (HRP) was added to each well and incubated for 1 h at room temperature. Lastly, tetramethyl benzidine (TMB) was added and incubated for 45 min. The reaction was stopped by adding stop solution (0.25 M sulfuric acid). The absorbance values were measured at 450 nm and the absorbance at the reference wavelength of 650 nm was subtracted using a molecular devices SPECTRAMAX 340 PC plate reader.

### Residual CHO host cell protein assay

The quantification of residual CHO host cell proteins (HCP) in the end product was performed by utilizing a Cygnus Technologies CHO HCP kit. The antibody coated microtiter strips provided with the kit were not used, and instead a Cygnus technologies polyclonal goat anti-CHO lysate coating antibody was purchased and used to coat a plate at 4 µg/mL in PBS (pH 7.4). The plate was coated overnight at 5 °C. A standard curve concentration of 1250 ng/mL to 4.88 ng/mL was used. The samples were diluted and combined with HRP-labeled goat anti-CHO conjugate antibody and mixed for 2 h at room temperature. The plate was simultaneously blocked with PBS/BSA for 2 h at room temperature. The standards, samples, and appropriate controls were then added to the plate and incubated for 2 h at room temperature. TMB substrate was added and incubated for 20 min protected from light at room temperature. Finally, the reaction was stopped using 1.0 M HCl. The absorbance values were measured at 450 nm and the absorbance at the reference wavelength of 650 nm was subtracted using a molecular devices SPECTRAMAX 340 PC plate reader.

### Residual DNA assay

CHO DNA in the cell-free harvest and downstream intermediates were quantified using real time q-PCR assay. DNA from the process intermediates, spiked samples (Post VI and final UF/DF intermediates for each of the homodimers were spiked to a level of 1000 pg/mL with CHO cell genomic DNA (Pacific Bio, Catalog # 1202-5) and controls were first extracted using Qiagen’s DNeasy Blood and Tissue Kit, which used minispin columns after the samples were pretreated with Proteinase K and carrier RNA. Samples were applied to the silica-based matrix and then washed with buffer three times prior to eluting the bound DNA into a 10 mM Tris-HCl, 1 mM disodium EDTA buffer, pH: 8.0 (Fluka). A set of CHO DNA standards ranging in concentrations from 10 to 100 000 pg/ml was prepared in nuclease-free water from a stock CHO DNA solution by serial dilution. All the samples were then mixed with the CHO forward and reverse primers, a probe labeled with a fluorescent reporter dye FAM (6-carboxyfluorescein) at the 5′ end and a quencher dye TAMRA (6-carboxy-tetramethylrhodamine) at the 3′ end and Taqman Mastermix. Each of these samples were then loaded onto a 96-well plate and analyzed in triplicate. qPCR reactions were performed and analyzed on an ABI Prism 7900 Sequence Detection System. TaqDNA polymerase begins amplification of the target DNA and upon reaching the probe, its 5′ nuclease activity cleaves the probe, resulting in the release of the reporter dye. With the reporter dye no longer in proximity to the quencher dye, the reporter fluoresces and this fluorescence intensity is directly proportional to the amount of PCR product accumulated. The PCR cycle during which the system begins to detect fluorescence is defined as the threshold cycle number (C_T_). The more CHO DNA present in the sample, the earlier the threshold cycle is reached. A standard curve is generated by plotting the C_T_ against the log_10_ concentration of the DNA standard and a straight line fit is obtained using linear regression analysis. This plot is then used to estimate the amount of DNA in the unknown samples.

### Residual 2-MEA assay

To monitor the efficiency of 2-MEA removal during diafiltration, residual 2-MEA was tested. The method includes release of thiols from the samples by tris(2-carboxyethyl)phosphine (TCEP) reduction, labeling of the released thiols with 6-aminoquinolyl-N-hydroxysuccinimidyl carbamate (AccQ-tag Ultra Derivatization kit, Waters), and analysis of the labeled thiols by RP UPLC. A standard curve for 2-MEA (Sigma/Fluka) in the presence of 1 mM TCEP was prepared to ensure full reduction of any potential oxidized 2-MEA (cystamine) impurity. The 2-MEA was separated on a AccQ-Tag Ultra C18 1.7 µM 2.1 × 100 mm column running at 55°C at 0.7 mL/min. Elution was performed using the following gradient conditions starting at 1% B (eluent A: Milli-Q water with 0.1% TFA) to 50% B (eluent B: 90% acetonitrile, 9.9% Mill-Q water and 0.1% TFA) in 6 min and to 99% B at 7 min. Labeled 2-MEA detection was achieved by measuring the fluorescence signals (i.e., Ex. 266 nm; Em 473 nm). A 7-point curve was generated by plotting thiol peak areas against corresponding known concentrations of derivatized standards of 2-MEA. A linear relationship between injected amounts of standards and the resulting UPLC peak areas was achieved from 0 to 50 μM with a correlation coefficient (r^2^) of the linear regression of 0.99. Samples were treated with 1 mM TCEP final concentration and centrifuged using Amicon Ultra-0.5 centrifugal filters. Ten microliters flow-through was further processed according to the kit labeling procedure (AccQ-tag Ultra Derivatization kit, Waters). Each sample was measured in triplicate.

### Dual-binding ELISA for the detection of EGFRxCD20 bispecific antibodies

The presence of EGFRxCD20 bispecific antibodies was determined using a sandwich ELISA as described previously.^33^ In short, ELISA plates (Greiner bio-one) were coated overnight with 2 µg/mL of recombinant EGFR (extracellular domain) in PBS at 4 °C. The plates were washed and incubated with serial diluted purified antibody samples diluted in PBS supplemented with 0.05% (v/v) Tween-20 and 0.2% (w/v) BSA (PBS-TB) for 90 min at 20 °C under shaking conditions (300 rpm). Next, the plates were washed and incubated with 2 µg/mL of mouse anti-idiotype monoclonal antibody 2F2 SAB1.1 (directed against HuMab-CD20; Genmab) diluted in PBS-TB for 75 min at 20 °C. Bound bispecific antibodies were detected with HRP-labeled goat-anti-mouse IgG (Jackson ImmunoResearch) and ABTS substrate (Roche Diagnostics). The color development reaction was stopped by addition of an equal volume of oxalic acid (Riedel de Haen) and absorbance was measured at 405 nm. Bispecific antibody samples were quantified by nonlinear regression curve-fitting (GraphPad Software) using a bench-scale EGFRxCD20 bispecific antibody as reference.

### Detection of Fab-arm exchange by electrospray ionization mass spectrometry (ESI-MS)

The presence of bispecific antibodies was determined using electrospray ionization mass spectrometry as described previously.^34^ Samples containing the antibody mixtures in 50 μL aliquots (200 μg/mL) were deglycosylated overnight with 1 μL N-glycosidase F (Roche Diagnostics). Samples were desalted on an Acquity UPLC (Waters) with a BEH300 C18, 1.7 μm, 2.1 × 50 mm column at 60 °C and 5 μL was injected and eluted with a gradient from 2% to 95% acetonitrile (LC-MS grade; Biosolve) in de-ionized water (Millipore) over a 5 min period. The gradient contained 0.1% formic acid as organic modifier (Fluka). ESI-TOF mass spectra were recorded online on a micrOTOF mass spectrometer (Bruker) operating in the positive ion mode. In each analysis, a 500–2 700 m/z scale was internally calibrated with ES tuning mix (Agilent). Mass spectra were deconvoluted using the Maximum Entropy algorithm provided in DataAnalysis software v3.4 (Bruker).

### Cation-exchange high pressure liquid chromatography (CIEX)

The efficacy of cFAE was assessed by analytical cation exchange liquid chromatography (CIEX). For this, samples were diluted to 2 mg/mL in eluent A (10 mM sodium phosphate, pH 7.0) and 25 μL was injected onto an Alliance 2795 HPLC separation module (Waters). The IgG molecules were separated based on charge using a ProPac^®^ WCX-10 column 4 × 250 mm (Dionex Corp.) with a flow rate of 1 mL/min at 30 °C. Elution was performed with a gradient to buffer B (10 mM sodium phosphate with 0.25 M sodium chloride, pH 7.0) using the following gradient conditions: 0% B initial 3 min to 72% B in 55.5 min. Next, 100% eluent C (10 mM sodium phosphate with 0.75 M sodium chloride, pH 7.0) for 5 min to clean the column. Protein elution was monitored by measuring the absorbance at 280 nm with a 2478 dual λ absorbance detector (Waters).

### SDS-PAGE

SDS-PAGE was performed to test the homogeneity and the purity of the stored samples. Both non-reduced and reduced SDS-PAGE was performed using the NuPAGE^®^ Bis-Tris electrophoresis system. Samples were diluted in LDS sample buffer, heated at 70 °C for 10 min and loaded on pre-cast 4–12% Bis-Tris gels. The gels were stained with Coomassie (Simply Blue Safe-Stain) and digitally imaged using the Optigo Imaging System (Isogen Life Science). Relative band areas were determined by gel scanning densitometry resulting from analysis of the Optigo images with TotalLab software.

### CE-SDS (reduced and non-reduced)

Purity and fragmentation of the samples was analyzed using CE-SDS on the Labchip GXII (Caliper Life Sciences). Sample preparation was performed with the HT Protein Express Reagent Kit according to manufacturer’s instructions (High Sensitivity protocol) with few modifications. Samples were prepared in 96-well Bio-Rad HSP9601 plates. Both non-reduced and reduced analysis (addition of DTT) was performed. Samples were denatured by incubation at 70°C for 10 min. The Chip was prepared according to manufacturer’s instructions and the samples were run with the HT antibody analysis 200 high sensitivity settings. Data were analyzed for kilodaltons and percent purity with Labchip GXII software.

### High performance size exclusion chromatography (HP-SEC)

Antibody batches were analyzed for monomer, multimer, and degradation product content by HP-SEC. For this, 50 μL antibody samples (IgG concentration between 1 and 10 mg/mL) were injected into an Alliance 2795 separation unit (Waters) connected to a TSK G3000SW_xl_ column (Tosoh Biosciences) pre-equilibrated with PBS and kept at 27.5 °C. The samples were run at 1 mL/min for 20 min. Protein detection occurred at 280 nm with a 2487 dual λ absorbance detector (Waters). Chromatograms were analyzed using Empower software, version 2002 (Waters), and expressed per peak as percentage of total peak area.

### N-linked glycan profiling 2-AB NP-HPLC

N-linked oligosaccharides were released by incubation with recombinant peptide-N-glycosidase F (Prozyme). Subsequently, the proteins were ethanol precipitated and removed. The supernatants were vacuum-dried. The oligosaccharides were labeled with the fluorophore 2- aminobenzamide (2-AB) label on the reducing end by reductive amination for HPLC analysis according to the manufacturer’s instructions provided in the LudgerTag™ 2-AB Glycan Labeling Kit. NP-HPLC profiles were obtained with a gradient elution and fluorescence detection as described previously.^29^

### Circular dichroism

A Jasco J-810 spectropolarimeter was used for CD measurements. The Far UV CD measurements were measured in a 0.1-cm path length cell. The IgG concentration was approximately 1 mg/mL in PBS. The far-UV CD spectra were recorded from 250 nm to 320 nm. For near-UV CD measurements, a cuvette of 1-cm path length was used and spectra were recorded from 190 nm to 260 nm. For both far- and near-UV CD measurements, each measurement was the average of three repeated scans with a scan speed of 20 nm/min (step resolution 0.1 nm, 1 s each step) from which the corresponding background was subtracted.

### Stability: bsIgG1-EGFRxCD20 formulation

The bsAb material obtained from the manufacturing-scale exchange was stored in PBS pH 7.4 (10 mM sodium phosphate, 140 mM sodium chloride). To test stability in other buffers, portions of the original batch were formulated to an IgG concentration of 14–15 mg/mL in each of 5 solutions: (a) PBS pH 7.4 by dilution with PBS; (b) PBS/2.5 mM EDTA pH 6.9 by addition of an EDTA stock solution (as the EDTA stock solution was not titrated, this resulted in a pH shift to 6.9); (c) PBS pH 6.0 by titration with 1 M phosphoric acid; (d) 30 mM histidine, 225 mM sorbitol pH 6.0 by buffer exchange using a microspin G-25 column (GE Healthcare); (e) 30 mM acetate, 225 mM sorbitol, pH 5.4 by buffer exchange using a microspin G-25 column (GE Healthcare). All the samples for each time point were aliquoted in separate glass vials with caps. For the samples in buffers a, b, and c, 500 µL volumes were aliquoted in HPLC grade glass vials (Waters P/N: 186000307C); for samples in buffers d and e, 300 µL was aliquoted.

### Stability: Comparison of parental antibodies, bsAb, and IgG1-EGFR

Parental mAbs IgG1–2F8-F405L and IgG1-7D8-K409R and bsAb were obtained as described above. The IgG1-EGFR was produced at the 10 000 L scale and purified using a downstream process that was similar to that described for the parental antibodies through the anion exchange process. Following anion-exchange, the IgG1-EGFR was further purified by bind/elute cation exchange chromatography and nanofiltration followed by ultrafiltration/diafiltration into a formulation buffer (50 mM phosphate, 50 mM NaCl, 3% (w/w) mannitol, 0.02% (w/v) polysorbate 80, 0.01% (w/v) EDTA, pH: 6.0). The IgG1-EGFR material was stored at 5 °C for 2 y until the start of this study. This panel of antibodies was formulated to a concentration of 13–14 mg/mL into different buffers using PD-10 desalting columns pre-packed with Sephadex G-25 resin (GE Healthcare) in a gravity flow mode. These buffers were chosen to be potentially favorable to promote: (1) deamidation (20 mM Tris, 20 mM NaCl, pH 8.5), (2) aggregation and/or fragmentation (40 mM sodium acetate, pH 4.0) and compared with the materials in PBS pH 7.4 as a reference. All samples for each time point were aliquoted in separate glass vials (Waters P/N: 186000307C) and incubated for up to 8 weeks at 25 °C.

## Supplementary Material

Additional material
