# Supplementary material for: Production of stable bispecific IgG1 by controlled Fab-arm exchange: Scalability from bench to large-scale manufacturing by application of standard approaches
Source: MAbs. 2013 Aug 22;5(6):962–73. doi: 10.4161/mabs.26233 (PMC3896610; doi:10.4161/mabs.26233)
Supplement: Additional material [file mabs-5-962-s01.pdf]

Landes Bioscience

www.landesbioscience.com

Supplemental Material to:

Michael J Gramer, Ewald TJ van den Bremer, Muriel D. van Kampen, Amitava Kundu, Peter Kopfmann, Eric Etter, David Stinehelfer, Justin Long, Tom Lannom, Esther H Noordergraaf, Jolanda Gerritsen, Aran F Labrijn, Janine Schuurman, Patrick HC van Berkel, and Paul WHI Parren

Production of stable bispecific IgG1 by controlled Fab-arm exchange: Scalability from bench to large-scale manufacturing by application of standard approaches

2013; mAbs 5(6)

http://dx.doi.org/10.4161/mabs.26233

http://www.landesbioscience.com/journals/mabs/article/26233/

Supplementary information for

Production of stable bispecific IgG1 by controlled Fab-arm exchange: scalability from bench to large scale manufacturing by application of standard approaches

Michael J. Gramer[3,4], Ewald T.J. van den Bremer[1], Muriel D. van Kampen[1], Amitava Kundu[3,4], Peter Kopfmann[3,4], Eric Etter[3,4], David Stinehelfer[3,4], Justin Long[3,4], Tom Lannom[3,4], Esther H. Noordergraaf[1], Jolanda Gerritsen[1], Aran F. Labrijn[1], Janine Schuurman[1], Patrick H.C. van Berkel[1,2], Paul W.H.I. Parren[1*]

[1]Genmab, Yalelaan 60, 3584 CM Utrecht, the Netherlands

[2]Current Address: ADC Therapeutics, Chemin de la Pacottaz 1, 1806 St-Legier, Switzerland

[3]Genmab, 9450 Winnetka Ave N, Brooklyn Park, MN 55445, USA

[4]Current Address: Baxter Healthcare, 9450 Winnetka Ave N, Brooklyn Park, MN 55445, USA

*Corresponding Author

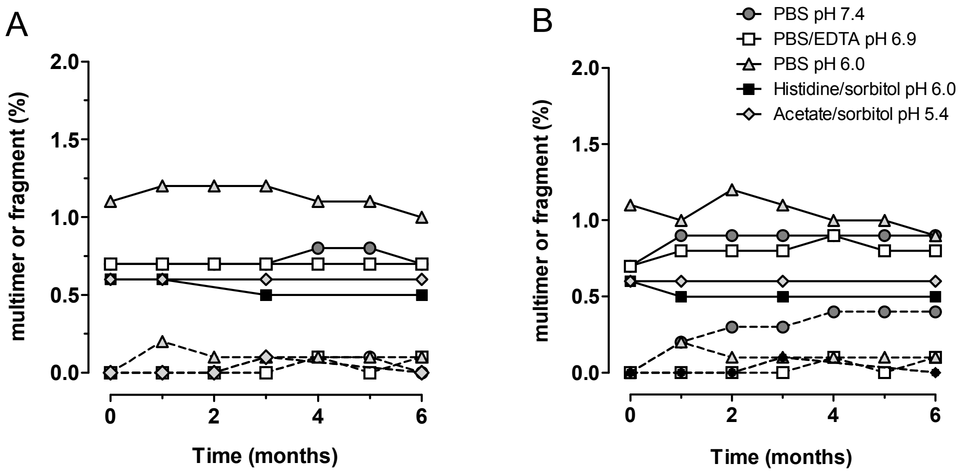

Supplementary Figure 1. Stability of bsIgG1-EGFrxCD20 in different buffers as analyzed by HP-SEC. Percentage of multimers (solid line) and fragments (dotted line) for bsIgG1-EGFRxCD20 upon storage during 6 months at 5°C (A) and 25°C (B). The PBS pH 6.0 formulation was prepared by titrating the PBS pH 7.4 batch with HCl. This procedure induced multimer formation, resulting in an increased start level of multimers of the bispecific material in PBS pH 6.0, but remaining constant during the course of the stability study. Also for the other formulations, the percentage of multimer did not change for either storage temperature. Fragmentation was slightly increased in PBS pH 7.4 at 25°C, whereas no detectable fragmentation could be observed in the remaining buffers.

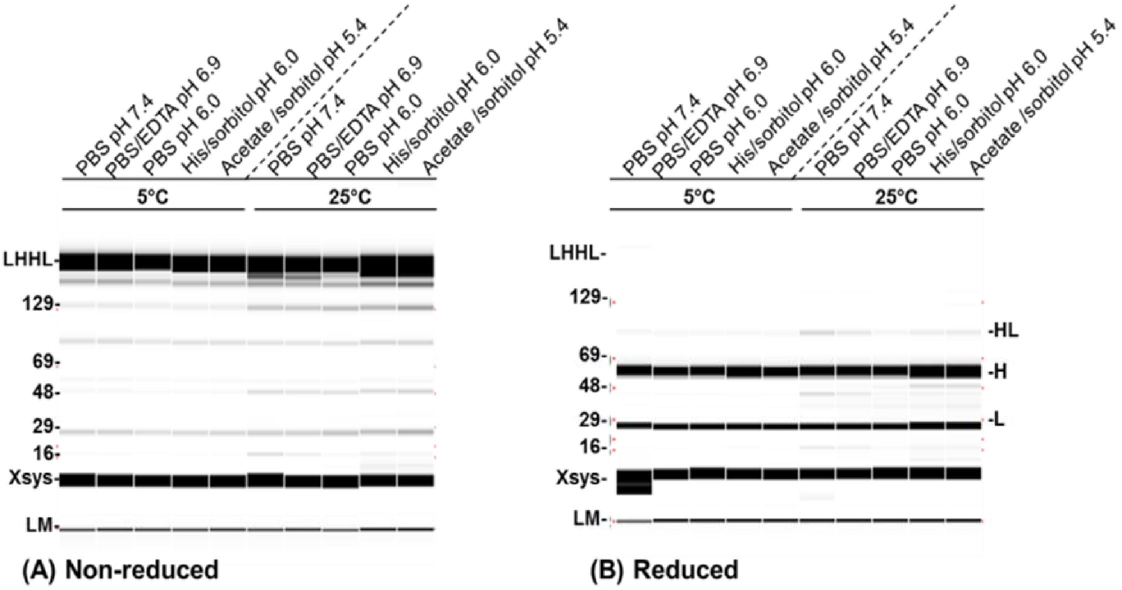

Supplementary Figure 2. Stability of bsIgG1-EGFRxCD20 for 6 months in different buffers as analyzed by non-reduced CE-SDS (A) and reduced CE-SDS (B). System peaks (Xsys), lower marker (LM), intact (LHHL), light chain (L), heavy chain (H), half molecule (HL) and molecular weights markers (kDa) are indicated. The band patterns at t=0 for the bispecific material in the different formulations (data not shown) were comparable to the patterns observed after storage for t=6 months at 5°C; The percentage of intact IgG was 97±1% under these conditions and the sum of the percentages of Heavy chain + Light chain under reduced conditions was 99±1%. The temperature increase to 25°C induced fragmentation and the formation of additional bands on reduced CE-SDS, with the lowest impact for the material in the PBS pH 6.0 formulation.

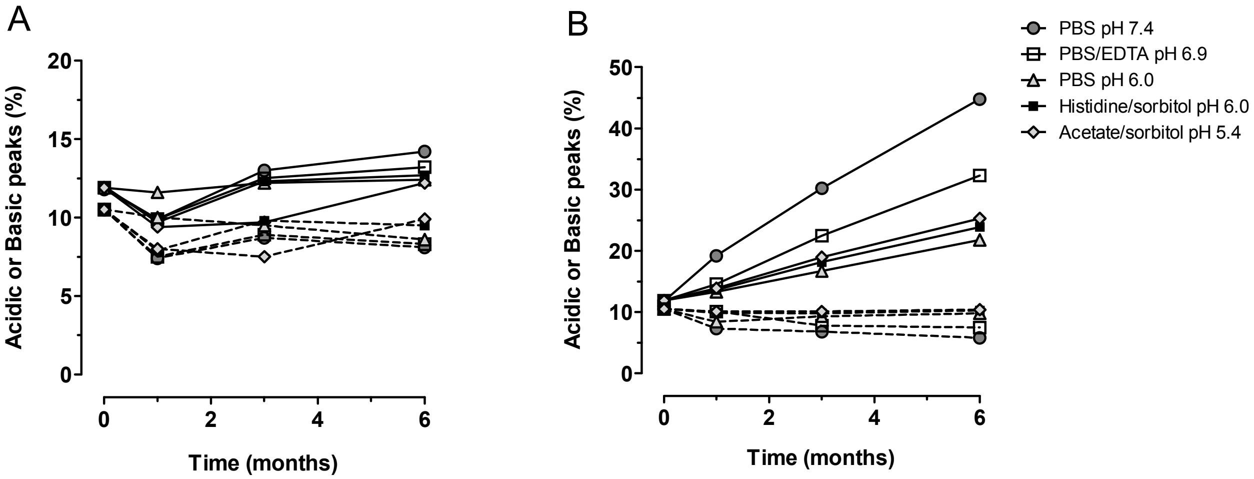

Supplementary Figure 3. Stability of bsIgG1-EGFRxCD20 in different buffers as measured by CIEX. Percentage of Acidic peaks (solid line) and Basic peaks (dotted line) for bsIgG1-EGFrxCD20 upon storage during 6 months at 5°C (A) and 25°C (B). No significant change in charged isoform distribution was observed upon storage at 5°C for 6 months. The higher temperature of 25°C induced an increase in the percentage of acidic peaks probably due to deamidation of the material. The deamidation rate was reduced in buffers with slightly acidic pH compared to PBS pH 7.4 buffer.
